# Cardiac Tamponade in Disguise: Purulent Pericarditis as a Deadly Turn in Pneumococcal Sepsis

**DOI:** 10.7759/cureus.93760

**Published:** 2025-10-03

**Authors:** Carla Gomes, Antonio G Novais, Rui Terras Alexandre, Carla Rebelo, Cristiana Teles, João Preto, Cristina Nunes, Domingos Fernandes

**Affiliations:** 1 Department of Intensive Medicine and Emergency, Northeast Local Health Unit, Bragança, PRT

**Keywords:** invasive, pericardial effusion, pericarditis, pneumococcal, pneumonia, tamponade

## Abstract

Invasive pneumococcal disease, presenting as purulent pericarditis, is a rare but potentially fatal condition. Early recognition and targeted antimicrobial and supportive interventions are essential to improve outcomes, particularly in high-risk patients.

A 62-year-old male with poorly controlled type 2 diabetes mellitus was admitted with community-acquired pneumonia, following a five-day history of respiratory symptoms prior to hospitalization. A thorax CT scan revealed left lower lobe consolidation and bilateral pleural effusion. Empiric antibiotic therapy was initiated with ceftriaxone and azithromycin. As transthoracic echocardiography showed pericardial effusion and due to signs of myopericarditis, colchicine and lysine acetylsalicylate were added to the treatment. On the second inpatient day, he developed respiratory failure and hemodynamic instability with atrial fibrillation. A transthoracic echocardiography was repeated, which showed worse pericardial effusion with signs of cardiac tamponade. Emergent ultrasound-guided pericardiocentesis was performed with purulent drainage. An immediate hemodynamic improvement was assessed. *Streptococcus pneumoniae* was isolated from blood and sputum cultures, with sensitivity to the initial antibiotics. The pericardial fluid showed intense neutrophilic inflammation, though culture was negative. The patient recovered without surgical intervention and was discharged after 14 days of antibiotics, four weeks of corticosteroids, and three months of colchicine. No signs of constrictive disease were observed on the follow-up medical appointment.

This case highlights the importance of vigilance for cardiac complications in invasive pneumococcal disease. In the presence of bacteremia and pericardial effusion, echocardiographic monitoring is crucial, as even small effusions can rapidly progress to tamponade. Prompt drainage, adequate antimicrobial therapy, and multidisciplinary care are key to successful outcomes.

## Introduction

Pericardial effusion may develop in the setting of pericarditis or arise as a "paraphenomenon" of uncertain significance in various systemic disorders. The etiology of pericardial effusion is idiopathic in approximately 29% of cases and may be related to iatrogenic causes (16%), neoplasia (13%), post-myocardial infarction (8%), uremia (6%), connective tissue and thyroid diseases (5%), or infection (2%) [1].

Pneumococcal invasive disease is a rare condition associated with a high mortality rate. Being lately diagnosed, usually in an advanced stage, it prompts a dreadful prognosis [2-4].

*Streptococcus pneumoniae* is the most common etiologic agent of community-acquired pneumonia and is typically covered with empiric antibiotic therapy. Some populations are most predisposed to associated complications, such as individuals with diabetes mellitus, chronic alcoholism, or immunosuppression. Despite being rare, pericarditis and pericardial effusion stand out among the most potentially serious complications [5].

## Case presentation

A 62-year-old man, with a history of poorly controlled type 2 diabetes mellitus and no record of pneumococcal vaccination, was admitted with five-day symptoms of community-acquired pneumonia. Blood analysis showed elevation of inflammatory parameters (Table 1), and chest CT scan revealed left inferior lobe consolidation and bilateral pleural effusion.

**Table 1:** Laboratory data of the patient on admission (day one) and on the day of cardiac tamponade (day two).

Laboratory sample			
Blood plasma			
Category, unit	Reference range	Day 1	Day 2
Hemoglobin, g/dL	14.0-17.5	14.7	12.5
Leukocytes, 10^9^cells/L	4.4-11.3	34.14	31.55
Neutrophils, 10^9^cells/L	1.5-7.5	30.38	28.45
Neutrophils, %	40-70	88.9	90.2
Lymphocytes, 10^9^ cells/L	1-4.8	0.98	1.03
Platelet count, 10^9^/L	150-450	252	272
High-sensitive troponin, ng/L	<20	160	825
Creatine kinase, U/L	<24	30	48
Lactate dehydrogenase, U/L	<248	295	133
C-reactive protein, mg/dL	<0.1	38.45	17.74
Procalcitonin, ng/mL	<0.5	11.59	6.88
Pericardial effusion			
Category, unit	Reference range	Day 1	Day 2
Leukocyte count, cells/mm^3^	<100	-	103528
Neutrophils, %	N/A	-	95
Lymphocytes, %	N/A	-	5
Glucose, mg/dL	>60	-	<10

Transthoracic echocardiography demonstrated pericardial effusion without signs of complication. Given its presumed association with systemic inflammation, a watchful waiting strategy was adopted. Empiric antibiotic therapy with ceftriaxone and azithromycin was initiated.

There was a slight increase in high-sensitivity troponin to 246 ng/L, and the electrocardiogram showed elevation of the ST segment in V1 derivation, which was assumed to be due to myopericarditis; for that reason, the patient was initiated on colchicine (1 mg per day) and lysine acetylsalicylate (1800 mg per day).

One day after admission, the patient developed respiratory failure and the need for invasive mechanical ventilation, along with progressively worsening hemodynamic status with escalating vasopressor support. By the end of the day, recurrent episodes of atrial fibrillation with rapid ventricular response and hemodynamic instability led to electrical cardioversion, which failed to produce a sustained effect. The patient progressed to refractory shock despite cardiovascular interventions.

**Figure 1:**
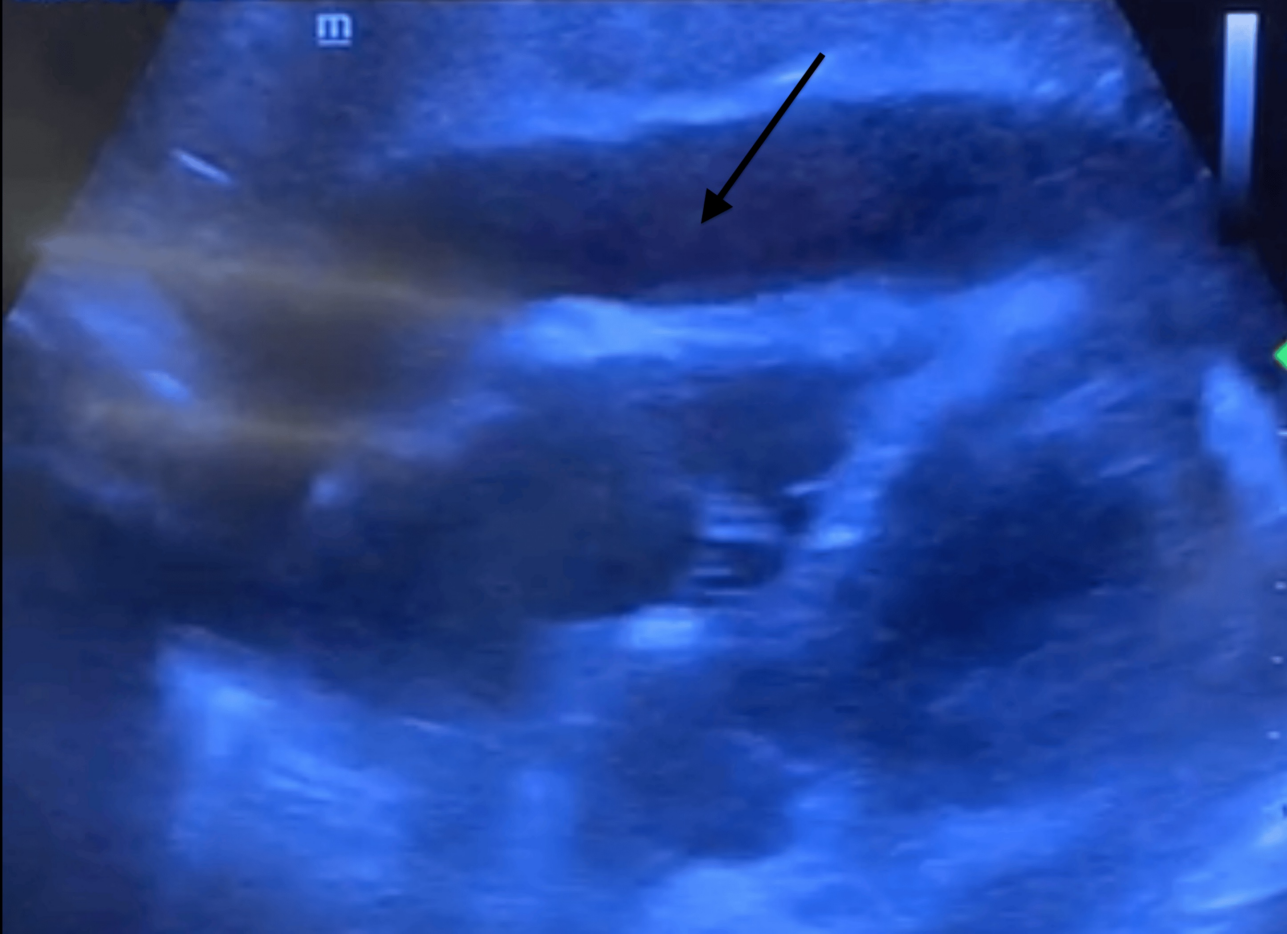
Transthoracic echocardiogram (subcostal view) showing pericardial effusion (marked with the arrow). Image captured from a video showing pericardial effusion with cardiac tamponade.

Transthoracic echocardiography showed a concentric pericardial effusion with a maximum width of 2.7 cm, with right chambers compression, transvalvular mitral flow with variability superior to 25%, and an inferior vena cava variation less than 20%, suggesting cardiac tamponade (Figure 1).

**Figure 2:**
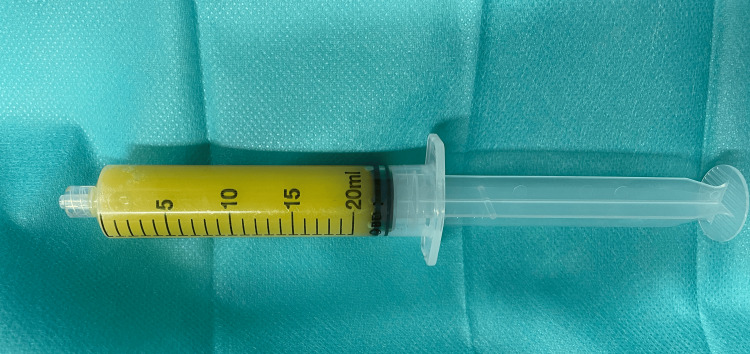
Syringe with purulent pericardial effusion drainage.

An obstructive shock secondary to cardiac tamponade was diagnosed, and the absence of response to any of the adopted measures (fluid therapy and vasopressor support) culminated in a “peri-arrest” period. Emergent ultrasound-guided pericardiocentesis was performed with drainage of 200cc of purulent content (Figure 2), and an immediate improvement of hemodynamic status was seen.

*Streptococcus pneumoniae* was isolated in sputum and blood cultures drawn at admission, with sensitivity to empiric antibiotic therapy in the course. Pericardial effusion with 103528 leucocytes per mm^3^ and 95% neutrophils, with direct visualization of gram-positive bacilli and cocci, but no bacterial growth in microbiological culture was noted.

On the etiological investigation of the pericardial effusion, all the conducted studies were negative, including polymerase chain reaction for *Mycobacterium tuberculosis* and culture for bacteria, fungi, and acid-fast bacilli.

Following consultation with the cardiothoracic surgical team, debridement was considered unnecessary, given the effectiveness of drainage. After drainage, clinical stability was accomplished, and the patient was successfully extubated six days after the event.

A control echocardiogram showed increased pericardial echogenicity, and it was decided to complete 14 days of antibiotherapy, four weeks of corticosteroid, and three months of colchicine. At the follow-up medical appointment, three months later, there was no evidence of constrictive disease or pericardial effusion.

## Discussion

Purulent pericarditis is defined as an infection in the pericardial space that produces macroscopically or microscopically purulent fluid. Once a common condition, purulent pericarditis is now a rarity with an estimated incidence of 1/18,000, in part due to the availability of effective antimicrobials [6].

Invasive pneumococcal disease is a rare diagnosis, especially presenting with purulent pericarditis. Timely recognition and the institution of an appropriate therapeutic approach are the pillars of successful management of this rare but potentially lethal condition.

The increasing prevalence of multi-resistant microorganisms may lead to a rise in this complication in the future [5,6]. The admission of these patients to the intensive care unit allows a rapid and effective recognition and resolution of a life-threatening situation. Despite appropriate antibiotic therapy, purulent pericarditis may require source control. In cases of cardiac tamponade, drainage and lavage might be necessary [2,7]. A study performed in animals showed penicillin G pericardial levels were already high after 30 minutes, and after 48 hours, no difference was found between the pericardial fluid and blood [8].

In the presence of bacteremia by pneumococcus, an echocardiogram must be performed, and in cases of pericardial effusion, progression must be monitored, since a small quantity of liquid can cause tamponade, and the clinical and echocardiographic vigilance allows a planned intervention when adequate. Pericardial constriction is a possible complication that must be closely monitored and prevented [9].

## Conclusions

Purulent pericarditis caused by *Streptococcus pneumoniae* remains a rare but potentially life-threatening condition in the era of antibiotics. Early recognition and prompt initiation of appropriate therapy are critical to improve patient outcomes. Optimal therapy includes targeted antibiotic treatment, hemodynamic stabilization, and continuous clinical and echocardiographic monitoring. Timely invasive interventions such as pericardiocentesis or surgical drainage may be required to achieve effective source control and prevent other complications. Continuous follow-up is essential to detect and prevent long-term sequelae. This case highlights the importance of maintaining a high level of suspicion for purulent pericarditis in patients with pneumococcal bacteremia and pericardial effusion, even in the current era of advanced antimicrobial coverage.

## References

[1] Sagristà-Sauleda J, Barrabés JA, Permanyer-Miralda G, Soler-Soler J (1993). Purulent pericarditis: review of a 20-year experience in a general hospital. J Am Coll Cardiol.

[2] Cronier P, Eugène B, Passefort S, Gryman R (2012). A pneumococcal purulent pericarditis revealing a pneumonia and complicated by an acute cardiac tamponade. J Cardiol Cases.

[3] Rees MJ, Wilson A (2019). Purulent pneumococcal pericarditis, a vaccine-preventable illness. Oxf Med Case Reports.

[4] Cillóniz C, Rangel E, Barlascini C, Piroddi IM, Torres A, Nicolini A (2015). Streptococcus pneumoniae-associated pneumonia complicated by purulent pericarditis: case series. J Bras Pneumol.

[5] Saenz RE, Sanders CV, Aldridge KE, Patel MM (1998). Purulent pericarditis with associated cardiac tamponade caused by a Streptococcus pneumoniae strain highly resistant to penicillin, cefotaxime, and ceftriaxone. Clin Infect Dis.

[6] Sagristà-Sauleda J, Mercé J, Permanyer-Miralda G, Soler-Soler J (2000). Clinical clues to the causes of large pericardial effusions. Am J Med.

[7] Thavendrarajah V, Ghia PS, Kozinn W, Little T (1993). Catheter lavage and drainage of pneumococcal pericarditis. Cathet Cardiovasc Diagn.

[8] Tan JS, Holmes JC, Fowler NO, Manitsas GT, Phair JP (1974). Antibiotic levels in pericardial fluid. J Clin Invest.

[9] Bass AN, Lynch S, Derr C, Gillen J (2025). A rapid point-of-care ultrasound diagnosis and treatment of tamponade in a patient with rare and lethal purulent pericarditis: a case report. Cureus.

